# Aplasia cutis congenita in monozygotic twins

**DOI:** 10.1002/ski2.270

**Published:** 2023-07-26

**Authors:** Hui‐Jun Lai, Mei‐Yan Lai, Ping‐Ping Ma, Hong‐Wei Guo

**Affiliations:** ^1^ The First Clinical Medical School Guangdong Medical University Zhanjiang Guangdong China; ^2^ Department of Dermatology Affiliated Hospital of Guangdong Medical University Zhanjiang Guangdong China

## Abstract

Aplasia cutis congenita (ACC) is defined as complete or partial loss or absence of skin at birth and it can occur on any part of the body, but most commonly on the scalp. Single offspring with ACC have been reported in most case reports, but cases in twins are rarely reported. Here, we report two cases of ACC, monozygotic twin boys presented with scattered skin absence over the scalp vertex after birth. All the lesions presented as ulcers with no hair and healed with scars, otherwise, the twins were well developed mentally and physically. In addition, the whole exome sequencing of the twins and their parents might provide diagnosis and classification assistance.

## 1 INTRODUCTION

**FIGURE 1:**
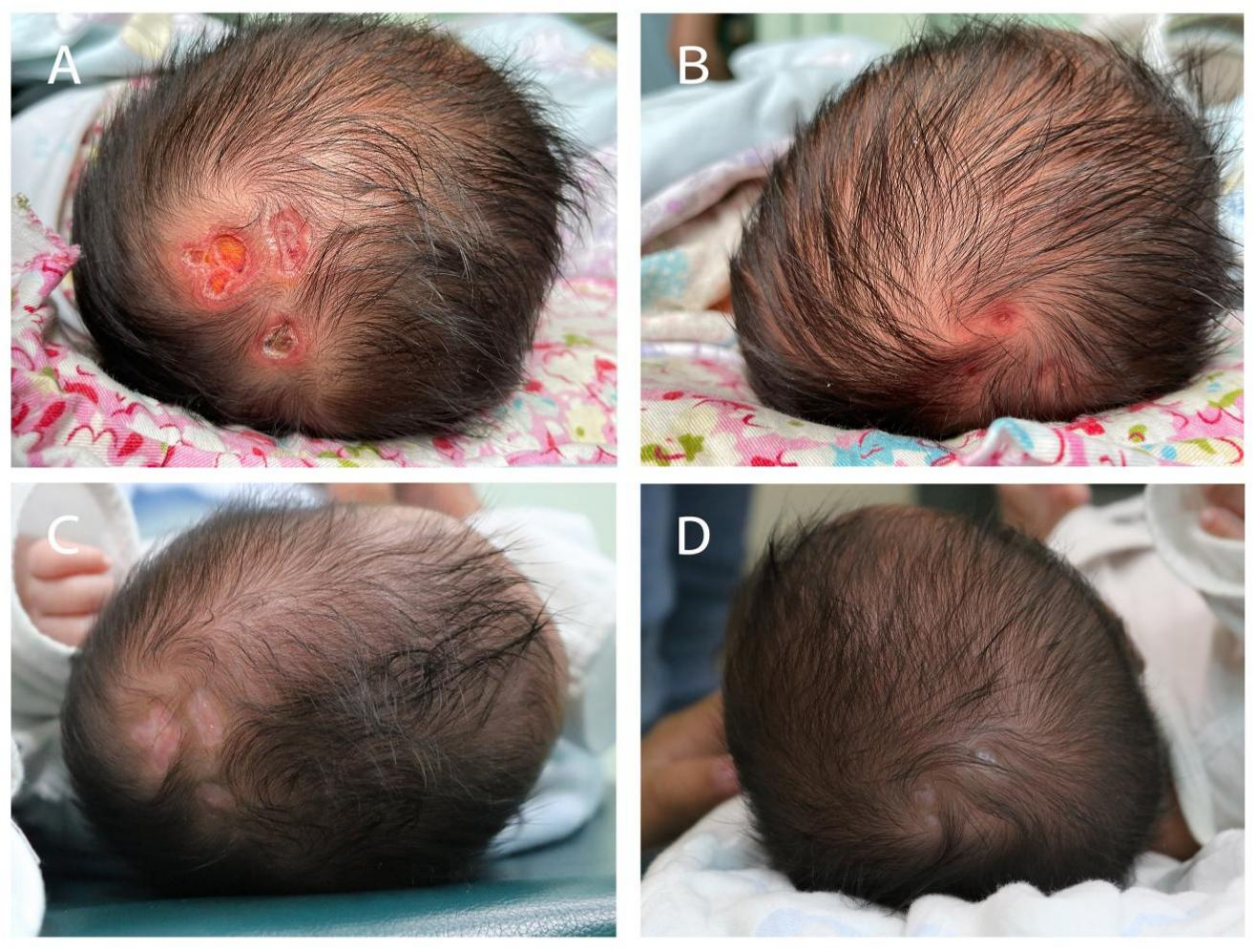
Two cases of aplasia cutis congenita in twins (a) and (b). Taken on the fifth day after birth, (a) Twin A presented with 2 × 2 cm, 2 × 1 cm and 1 × 1 cm scalp absence around his hair whorl. (b) Twin B was accompanied by three regions of circular skin absence about 0.8 cm in diameter around his hair whorl. (c) and (d) Photographs taken a month after birth, crusts fell off from the lesions of Twin A (c) and Twin B (d) leaving scar tissue and an absence of hair follicles.

Monozygotic twin boys, born prematurely after 35 weeks and 5 days of gestation to a 27‐year‐old woman via cesarean delivery, presented with scattered skin absence over the scalp vertex after birth. They were born 2 min apart (Twin A first) and Apgar scores were 10 and 10 at 1 and 5 min, respectively. Twin A, weight 2690 g, presented with 2 × 2 cm, 2 × 1 cm and 1 × 1 cm scalp absence around his hair whorl (Figure 1a). Twin B, with birth weight of 2660 g, exhibited three regions of circular skin absence about 0.8 cm in diameter around his hair whorl (Figure 1b).

All the lesions presented as ulcers with no hair, the shapes of which varied considerably including round, oval, irregular or “punched‐out” appearances. The bases of the ulcers were clear, crusts were seen on some ulcers with the depth of the ulcers little different. The rims of the ulcers were red while the boundaries of the ulcers were clear (Figure 1a,b). Cranial ultrasound was employed to check the completion of the cranial bones and any abnormality of the brain. It showed that the cranial bones underlying the lesions were intact and no vascular deformation were found. There were no other physical abnormalities such as nail loss and skin absences of the limbs identified.

**FIGURE 2:**
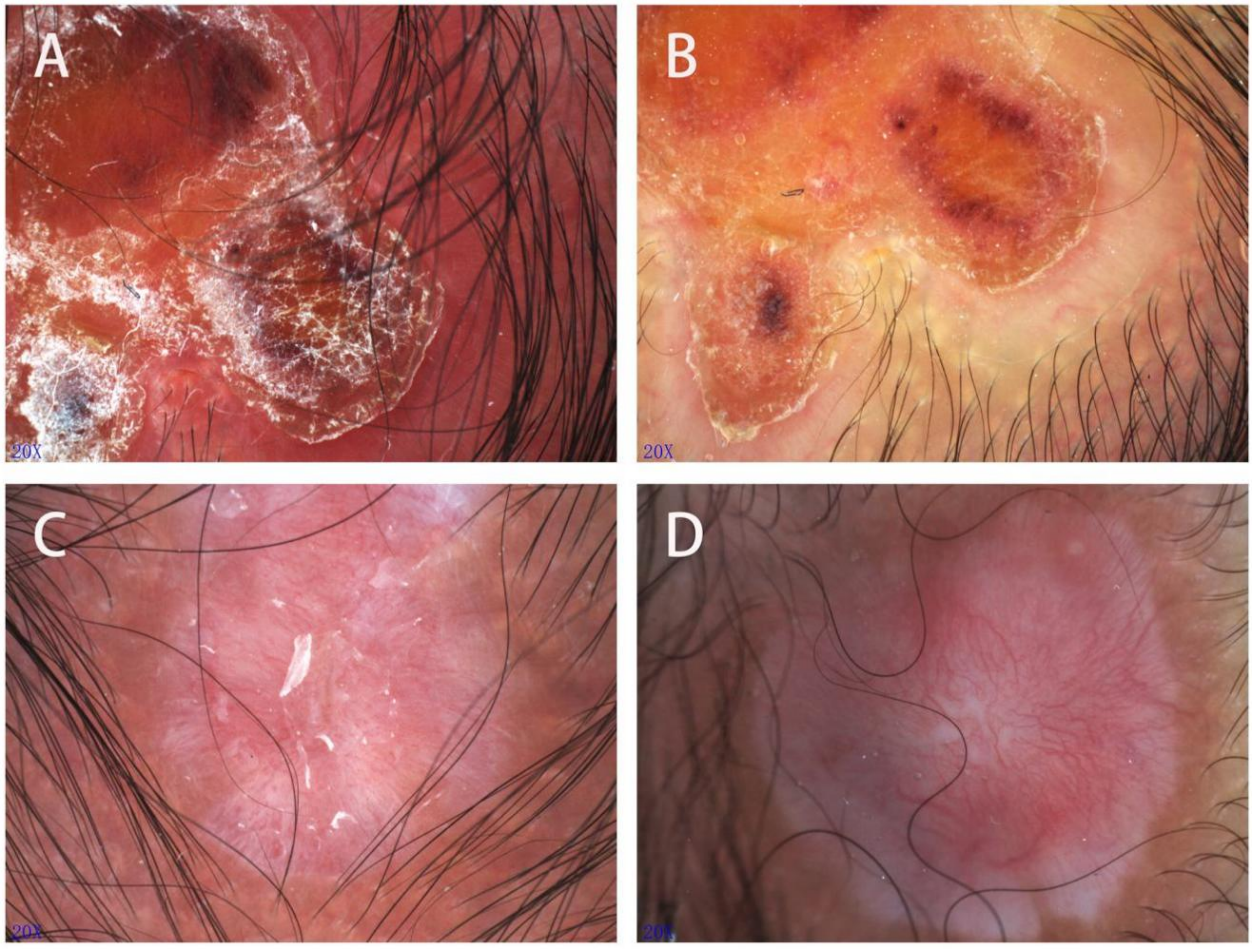
Dermoscopy of Twin A. (a) and (b) Taken on the fifth day after birth, showing brown‐yellowish ulcers on red‐yellowish backgrounds with abundant whitish scales and scattered purple‐black crusts. (c) and (d) Taken a month after birth, showing a few whitish scales (c) and abundant linear irregular telangiectasias (d) on yellow‐pinkish backgrounds, in conjunction with orange‐brown areas of pigmentation around the skin lesions (d). All images 20x.

On the fifth day after birth, dermoscopy was performed, and revealed brown‐yellowish ulcers on red‐yellowish backgrounds with abundant whitish scales and scattered purple‐black crusts (Figure 2a,b). One month after birth, as the crusts of the lesions fell off, the ulcers healed with scars (Figure 1c,d). Dermoscopy demonstrated a few whitish scales and abundant linear irregular telangiectasias on yellow‐pinkish backgrounds, in conjunction with orange‐brown areas of pigmentation around the skin lesions (Figure 2c,d).

The mother had gestational diabetes mellitus during pregnancy. A rapid plasma reagin test (RPR) for syphilis and a herpes PCR test were both negative. There was no maternal use of alcohol or drugs. Family history was unremarkable. The laboratory examination of the infants, including RPR and herpes PCR, did not show abnormalities as well. In addition, there was no positive family history, and whole exome sequencing results of the twins and their parents did not reveal a clear pathogenic variant that was clinically relevant to the subject. Based on the clinical manifestations and the Frienden classification,^1^ a diagnosis of aplasia cutis congenita (ACC), type 1 (scalp ACC without multiply anomalies or isolated ACC) was established.

## 2 DISCUSSION

ACC is defined as complete or partial loss or absence of skin at birth.^2^ It can occur on any part of the body, but most commonly on the scalp. ACC is an uncommon disorder with a birth rate of around 1 to 3 in 10 000 live births.^3^ Post wound healing, these scalp defects can easily be confused with other diseases of hair loss such as alopecia areata, scarring alopecia, trichotilomania, traction alopecia and syphilitic alopecia.

Although the exact pathogenesis of ACC has not been surely elucidated, the underlying factors could involve mutations in the genes associated with skin morphogenesis and keratinocyte proliferations and differentiations. The mutations of gene Ribosome biogenesis factor (BMS1),^4^ potassium‐channel tetramerization‐domain‐containing 1 (KCTD1), EOGT (endoplasmic reticulum‐resident O‐GlcNAc transferase), NOTCH1, RBP/J, Cdc42 and Rac1have been revealed in ACC.^5^ Forme‐fruste of neural tube defects,^6^ incomplete closure of the embryonic fusion lines, amniotic membrane adhesions, intrauterine infections or trauma, fetus papyraceus^7^ (dead fetus in utero caused by ischemia or thrombotic events), and teratogens were reported in ACC as well. A variety of drugs including methimazole,^8^ benzodiazepines,^9^ valproic acid,^10^ angiotensin converting enzyme inhibitors, cocaine and heroin,^11^ were also implicated in the development of ACC. In the cases presented here, the above‐mentioned causes of ACC could not be identified, therefore, we assume that the variation in blood flow caused by placental insufficiency, which might be related to gestational diabetes mellitus, may have played a role in the development of the ACC.

Nine subtypes Frieden classification of ACC has been wildly used on clinical grounds according if the lesions are complicated with other anomalies or the location of lesions.^1^ The case here would be type 1 (scalp ACC without multiply anomalies or isolated ACC) giving that the lesions were only located in the scalp and no other abnormalities were found.

The preferred therapy options include conservative treatment and surgery for aesthetic purposes.^12^ There is a slight risk of central nervous system infection for minor defects in the scalp without skin areas before healing. Therefore, small lesions are cleaned with normal saline and anti‐infective treatments, and no further treatment is required after the defects are healed. However, some larger defects can include skull defects or underlying bone defects that require the patients to undergo repair surgery. Surgical methods include skin flaps, skin allografts, skin grafts and biological or synthetic skin substitutes.^13^ For the case of the twins here, Ethacrdine lactate monohydrate solution was applied to the lesions twice a day, the ulcers gradually healed with scar.

Single offspring with ACC have been reported in most case reports, but cases in twins are rarely reported. Physicians should consider that these infants may have other congenital defects. However, in the case reported here, no other congenital defects have been found so far.

## CONFLICT OF INTEREST STATEMENT

The authors declare no conflicts of interest.

## AUTHOR CONTRIBUTIONS

**Hui‐Jun Lai**: Data curation (equal); writing—original draft (equal). **Mei‐Yan Lai**: Data curation (supporting); writing—review & editing (supporting). **Ping‐Ping Ma**: Formal analysis (supporting); writing—review & editing (supporting). **Hong‐Wei Guo**: Formal analysis (equal); writing—review & editing (equal).

## ETHICS STATEMENT

We confirm that the study has been approved by research ethics committees of The second Affiliated Hospital of Guangdong Medical University and that appropriate consent has been obtained for studies involving human participants.

## Data Availability

Data sharing not applicable to this article as no datasets were generated or analysed during the current study.
